# Genetic and Epigenetic Targeting Therapy for Pediatric Acute Lymphoblastic Leukemia

**DOI:** 10.3390/cells10123349

**Published:** 2021-11-29

**Authors:** Huan Xu, Hui Yu, Runming Jin, Xiaoyan Wu, Hongbo Chen

**Affiliations:** Department of Pediatrics, Union Hospital, Tongji Medical College, Huazhong University of Science and Technology, Wuhan 430022, China; drxu_2020@hust.edu.cn (H.X.); wendyyuhuily@hotmail.com (H.Y.); rmjin@hust.edu.cn (R.J.)

**Keywords:** pediatric acute lymphoblastic leukemia, genomics, epigenetics, targeted therapy

## Abstract

Acute lymphoblastic leukemia is the most common malignancy in children and is characterized by numerous genetic and epigenetic abnormalities. Epigenetic mechanisms, including DNA methylations and histone modifications, result in the heritable silencing of genes without a change in their coding sequence. Emerging studies are increasing our understanding of the epigenetic role of leukemogenesis and have demonstrated the potential of DNA methylations and histone modifications as a biomarker for lineage and subtypes classification, predicting relapse, and disease progression in acute lymphoblastic leukemia. Epigenetic abnormalities are relatively reversible when treated with some small molecule-based agents compared to genetic alterations. In this review, we conclude the genetic and epigenetic characteristics in ALL and discuss the future role of DNA methylation and histone modifications in predicting relapse, finally focus on the individual and precision therapy targeting epigenetic alterations.

## 1. Introduction

Leukemia is the most common malignancy in children and adolescents, and is responsible for a third of childhood cancer deaths. Most childhood leukemias are acute lymphocytic leukemia (ALL), followed by acute myeloid leukemia (AML), and chronic leukemias are rare in children. ALL results from the clonal proliferation of lymphoid stem or progenitor cells, with more than 80% being originated from B-cell progenitors (B-ALL) [1]. Both B-ALL and T-ALL immunophenotype groups comprise multiple subtypes defined by chromosome alterations that are believed to be leukemia-initiating lesions. 

The treatment of ALL has been one of the great success stories in cancer treatment, mainly owing to multi-agent chemotherapy regimens, central nervous system (CNS) prophylaxis, extended maintenance regimens, and risk-adapted treatment strategies [2]. Despite cure rates of ALL exceeding 90% in children, the treatment of relapsed or drug-resistant leukemia and some molecular subtypes remains challenging, thus it is still an important cause of morbidity and mortality in children. With such high survival, there is little room for further improvement in outcomes based on increased treatment intensity without unacceptable toxicity. St. Jude’s Total 16 study found that the intensity of conventional chemotherapy has reached its limit [3]. Therefore, we need to combine or replace traditional highly toxic chemotherapy with molecular targeted therapy and immunotherapy in order to improve the cure rate of the disease and the quality of life of patients [4]. In recent years, second-generation sequencing technologies such as whole transcriptome sequencing (RNAseq) have identified many special genotypes that are of great significance to prognosis and treatment [1,5]. Current efforts are aimed at appropriately stratifying patients and identifying targetable genetic lesions that would allow for personalized and precise treatment [6]. 

Conventional karyotyping has historically been used to identify genetic abnormalities to diagnose and risk-stratify children with ALL. However, genomic studies have revolutionized our understanding of the molecular taxonomy of ALL and have been used to clarify the subclassification of ALL. Many of these genetic alterations have important implications for diagnosis and risk-stratification of ALL and for the development of novel and targeted treatments [7].

The term “epigenetics” refers to the changes in gene expression that are inheritable through cell division rather than caused by changes in the DNA sequence itself. Three systems, including DNA methylation, RNA-associated silencing, and histone modification, contribute to initiate and sustain epigenetic silencing [8]. Normal hematopoietic cell development requires tightly controlled regulation of DNA methylation, chemical modification of histones, and expression of non-coding RNA, all of which may be deregulated during leukemic transformation [9]. 

DNA methylation is by far the most well-characterized epigenetic modification. Methylation of the C^5^ position of cytosine residues in DNA to form 5-methylcytosine has long been recognized as an epigenetic silencing mechanism [10]. The methylation of CpG sites within the human genome is maintained by several DNA methyltransferases (DNMTs), and aberrant de novo methylation of CpG islands is a hallmark of human cancers and is found early during carcinogenesis [11]. Histone modifications have also been defined as epigenetic modifiers. Post-translational modifications of histones mainly include acetylation, methylation, phosphorylation, ubiquitination [12]. MicroRNAs are short single-strand non-coding RNA molecules, which can interfere with mRNA to negatively affect protein translation and function as both tumor suppressors and oncogenes, depending on the targeted gene [13]. 

Many studies have suggested that genetic and epigenetic alterations play an important role in the pathogenesis, treatment outcome and relapse of ALL. Here, we focus on the somatic mutational signatures and epigenetic abnormalities of ALL, especially DNA methylations and histone modifications, summarize and discuss the role of epigenetic alterations in predicting relapse and targeted therapy.

## 2. Genetic and Epigenetic Characteristics of Pediatric ALL

### 2.1. B-Cell Acute Lymphoblastic Leukemia (B-ALL)

B-cell acute lymphoblastic leukemia is the most common form of ALL, and accounts for approximately 80–85% of pediatric ALL, resulting from arrest at an immature B-precursor cell stage. Although various environmental, ethnic, socioeconomic, infectious, immunological factors have been evaluated as potential contributors to leukemogenesis, the underlying etiologies of most cases of pediatric ALL remain unknown [14]. Most cases of B-ALL appear to arise spontaneously and are classified by the presence of recurrent somatic cytogenetic or molecular alteration [15]. The three major types of genetic alteration are chromosomal aneuploidy, rearrangements and point mutations (Table 1). Accumulating evidence suggests that the pathogenesis and phenotypic characteristics of leukemia are the results of the combination of specific targeted and genome-wide alterations of DNA methylation [16]. In addition, aberrant promoter methylation is associated with cytogenetic alterations [17], cytogenetic subtypes [18], prognosis [19], and relapse [20]. 

#### 2.1.1. High Hyperdiploidy (HeH)

High hyperdiploidy (51–67 chromosomes per leukemia cell) is a common subtype of pediatric ALL, and occurs in approximately 25% of childhood ALL [21]. HeH is characterized by the nonrandom gain of chromosomes 4, 6, 10, 14, 17, 18, 21, and X [22], and the most prominent epigenetic feature of HeH is a strong hypomethylation signature compared to the other ALL subtypes [9]. Paulsson et al. suggested that chromosomal gains were early driving events in HeH pathogenesis through whole-genome sequencing, and they found that HeH is associated with mutations in the Ras pathway, chromatin modifiers such as *CREBBP* [21]. The patients with high-hyperdiploid B-ALL have excellent outcomes, and the inferior clinical outcomes previously associated with low-hyperdiploidy (47–50 chromosomes) appear to be improved with contemporary therapy [23]. 

#### 2.1.2. Hypodiploidy

Hypodiploid B-ALL (less than 44 chromosomes) accounts for 1–2% of pediatric ALL and is associated with inferior survival, especially in those with end-of-induction minimal residual disease (MRD) positivity [24]. *TP53* mutations commonly occur in children with low-hypodiploid (30–39 chromosomes) ALL [25]. Low hypodiploidy (31–39 chromosomes) occurs in 1% of children with ALL but in more than 10% of adults. Holmfeldt et al. found that low hypodiploidy is characterized by the deletion of *IKZF2* and by near- universal *TP53* mutations and can be inherited in approximately half the patients [25]. Another chromosomal aneuploidy is near haploidy (24–30 chromosomes), which is present in approximately 2% of children with ALL and is associated with Ras mutations and deletions of *IKZF3*. The latter two chromosomal alterations are both associated with unfavorable outcomes [7].

#### 2.1.3. ETV6-RUNX1 Rearrangement

The *ETV6-RUNX1* fusion gene occurs in approximately 25% of standard-risk childhood B-ALL cases who have a t(12;21)(p13;q22), and is a favorable prognostic marker [24]. Greaves et al. suggested that *ETV6-RUNX1* translocations cooperated with additional necessary mutations to contribute to ALL pathogenesis [26]. *ETV6-RUNX1*-like ALL is characterized by a gene expression profile and immunophenotype (CD27^+^, CD44^low/negative^) similar to that of ALL with *ETV6-RUNX1* rearrangement [27,28]; this subtype occurs almost exclusively in children (approximately 3% of pediatric ALL) and is associated with relative favorable outcomes [29].

#### 2.1.4. KMT2A Rearrangement

Lysine-specific methyltransferase 2A (*KMT2A*) is a promiscuous gene with more than 80 different gene-fusion partners, which is also known as *MLL* (mixed-lineage leukemia) [30]. In addition, the somatic translocation of *KMT2A* occurs in approximately 75% of infants with B-ALL, especially in those <6 months of age [24], which comprise a distinct disease entity with an aggressive disease with poor prognosis [31]. Approximately 2% of older children, adolescents, and adults with ALL also have *KMT2A* translocation, and more than 100 fusion partners have been identified to date [32]. Infants with *KMT2A* rearrangement ALL have a remarkable paucity of other genetic abnormalities, but display typical DNA methylation profiles [33]. In addition, the DNA methylation pattern might underlie functionally relevant changes depending on the translocation partner of *KMT2A*. Pediatric ALL with *KMT2A* rearrangement are generally inferior to those of patients with non-*KMT2A* rearrangement ALL, and infants diagnosed at <90 days of age have a particularly dismal outcome [24].

#### 2.1.5. BCR-ABL1 Rearrangement

Philadelphia chromosome (Ph+) or t(9;22)(q34;q11.2) occurs in 3–5% of childhood B-ALL and nearly all patients with chronic myeloid leukemia (CML), which results in *BCR-ABL1* fusion gene [24]. *BCR-ABL1* fusion is a prognostic indicator of an advanced disease and a biomarker for targeted therapy with imatinib or dasatinib [34]. A multi-center randomized clinical study that we did in collaboration with St Jude Children’s Research Hospital showed that pediatric patients who received chemotherapy with dasatinib had better EFS, overall survival (OS), and CNS disease control when compared to patients who received imatinib [35]. *BCR-ABL1* fusion is the most difficult subtype of ALL to distinguish based on DNA methylation [36], thus the DNA methylation signatures need to be further clarified.

#### 2.1.6. TCF3 Rearrangement

*TCF3-PBX1* fusion gene results from the translocation t(1;19)(q23;p13.3) and occurs in approximately 4% of ALL cases, which is associated with an intermedia risk and more frequent CNS relapse [24]. Another rare fusion gene *TCF3-HLF* occurs in <0.5% of children with B-ALL, resulting from t(17;19)(q22;p13.3). In addition, *TCF3-HLF* fusion is associated with extremely poor outcomes [37].

#### 2.1.7. dic(9;20)

The chromosomal aberration dic(9;20)(p13.2;q11.2) occurs in up to 5% of B-ALL cases [38]. The translocation results in the loss of chromosome arms 9p and 20q and produces a fusion gene involving *PAX5* in some cases [39]. It is not yet known whether the oncogenic mechanism underlying the dic(9;20) subtypes is a gene fusion, loss of DNA from 9p and 20q, or a combination of both.

#### 2.1.8. iAMP21

Intra chromosome amplification of chromosome 21 (iAMP21) occurs in approximately 2% of childhood B-ALL and is more prevalent in older children, which was previously associated with a high risk of relapse and poor outcomes [40]. In addition, the prognosis of ALL with iAMP21 has improved with intensified treatment protocols [34]. The unifying feature of all iAMP21 cases is the amplification of the *RUNX1* locus on chromosome 21, and there is an overlapping signature between the iAMP21 and HeH cases [24].

#### 2.1.9. Philadelphia Chromosome-like ALL

*BCR-ABL1*-like or Philadelphia chromosome-like ALL is defined by an activated kinase gene expression profile similar to that of Ph+ ALL and associated with a diverse range of genetic alterations that activate cytokine receptor signaling pathways [41]. Ph-like subtype of pediatric ALL occurs in 10% of NCI standard risk and 13% of NCI high risk ALL cases [40]. Deletions and inactivating mutations of *IKZF1* and other lymphoid-associated transcription factors genes are common in Ph-like ALL [42]. In addition, children with Ph-like ALL have high incidences of treatment failure, relapse, and death when treated with conventional cytotoxic chemotherapy [43].

#### 2.1.10. Trisomy 21-Associated ALL

Children with trisomy 21 (Down Syndrome) have a 20-fold increased risk of developing ALL (also known as DS-ALL) [44]. In addition, DS-ALL is almost always B-lineage and has a lower incidence of hyperdiploidy and fewer recurrent cytogenetic translocations than in non-DS-ALL. Buitenkamp et al. reported that children with DS-ALL have an increased risk of chemotherapy-related toxicity and inferior survival [45]. The Philadelphia chromosome-like subtype of ALL is the most common form in DS-ALL. Kubota et al. reported that hypermethylation of *RUNX1* on chromosome 21 was found in DS-ALL, and they suggested that the hypermethylation of the *RUNX1* promoter in B-cell precursors might be associated with increased incidence of B-ALL in DS patients [46].

#### 2.1.11. DUX4 Rearrangement

*DUX4* (double homeobox 4) rearrangement was reported in up to 7% of childhood B-ALL cases and results in loss of function of *ERG* (*EST-related gene*) [24]. *DUX4* rearranged B-ALL has a distinct gene expression profile and immunophenotype (CD2^+^, CD371^+^) [47]. *ERG-DUX4* fusion has frequent concomitant *IKZF1* deletions (approximately 40% of cases), but also has excellent clinical outcomes with standard chemotherapy [48].

#### 2.1.12. MEF2D and ZN384 Rearrangements

ALL with *MEF2D* (myocyte enhancer factor 2D) rearrangement occurs in 3–6% of childhood B-ALL, more commonly in older children and adolescents, which may be associated with poor outcomes [24]. *MEF2D*-rearranged ALL has a distinct immunophenotype (CD10^−^, CD38^+^), and this rearrangement results in increased HDAC9 expression and sensitivity to histone deacetylase inhibitors [49,50,51]. *ZN384* (zinc finger protein 384) rearrangements have been described in approximately 3% of childhood B-ALL, and were associated with an intermediate prognosis [1]. Griffith et al. found that *ZNF384*-rearranged ALL is associated with elevated FLT3 expression [52]. In addition, Oberley et al. reported a case with *TCF3-ZNF384* fusion, and thought that this lineage-ambiguous phenotype may shift during the disease course and may result in loss of CD19 expression and failure of chimeric antigen receptor T(CART)-cell therapy [53].

#### 2.1.13. NUTM1 Rearrangements

*NUTM1* (nuclear protein in testis carcinoma family 1) rearrangements occur in 1–2% of pediatric B-ALL and is associated with excellent outcome, which could be fused to genes encoding various transcription factors and epigenetic regulators, such as *ACIN1*, *BRD9*, *IKZF1*, and *ZNF618*. *NUTM1* is supposed to result in global changes in chromatin acetylation and increased sensitivity to histone deacetylase inhibitors or bromodomain inhibitors [54].

### 2.2. T-Cell Acute Lymphoblastic Leukemia (T-ALL)

T-cell acute lymphoblastic leukemia (T-ALL) are immature lymphoid tumors localizing in the bone marrow, mediastinum, central nervous system, and lymphoid organs. They account for 10–15% of pediatric and about 25% of adult ALL cases. T-ALL arises in the thymus from an immature thymocyte as a result of a stepwise accumulation of genetic and epigenetic abnormalities (Table 1) [55]. Epigenetically, T-ALL is characterized by the gene expression changes caused by hypermethylation of tumor suppressor genes, histone modifications, and miRNA and lncRNA alterations [55]. Compared to B-ALL, T-ALL has a worse outcome, and the prognostic significance of recurrent T-ALL-associated mutations remains incompletely understood. Despite a growing understanding of genetic abnormalities in ALL, there are currently no other known reliable molecular genetic markers than the MRD for identifying patients with a higher risk of relapse specifically in T-ALL [55]. Risk stratification of patients with T-ALL is largely determined by CNS status and early response to therapy, which are measured by MRD testing [56].

#### 2.2.1. Number and Types of Chromosomal Abnormalities

Approximately 50% of cytogenetically abnormal pediatric T-ALL cases have only one chromosomal aberration [55]. Structural chromosome changes are much more common than numerical changes, and 90% of T-ALL with single chromosomal changes are structural and 10% numerical. Therefore, T-ALL is typically karyotypically characterized by the presence of only one or a few structural chromosomal aberrations [55].

#### 2.2.2. Recurrent Chromosome Translocations

The chromosomal translocations involving the fusion of T-cell receptor genes to oncogenes or interstitial deletions leading to the juxtaposition of two genes account for about 50% of pediatric T-ALL cases [24]. These chromosomal translocations result in altered expression of the transcription factors, subsequently leading to abnormal expression of genes involved in regulation of T cell development [57]. The genomic and epigenomic profiles studies have divided T-ALL into four major subtypes: (i) *TLX1* (T cell leukemia homeobox protein 1, previously termed *HOX11*), (ii) *LYL1*, (iii) *TAL1/LMO2*, and (iv) *TLX3* (previously termed *HOX11L2*), although the prognostic and therapeutic significance of the subtypes has not been well-elucidated [57]. Subsequently, a comprehensive genomic analysis of more than 260 pediatric and young adult T-ALL patients classified these patients into eight major groups based on the translocated gene and its dysregulated expression, including *TLX1*, *TLX3*, *TAL1*, *TAL2*, *LMO1/2*, *NKX2-1*, *HOXA*, and *LMO2-LYL1* [58].

More than 75 fusion genes have so far been reported in T-ALL, which are generated mainly through translocations, deletions, insertions [55]. Approximately 5–10% of pediatric T-ALL cases have *NUP214-ABL1* fusion resulting from t(5;14), and *KMT2A* rearrangement has been reported in 10–15% of T-ALL resulting from 11q23 [24]. *PICALM* (phosphatidylinositol binding clathrin assembly protein)-*MLLT10* (mixed-lineage leukemia; translocated to 10) fusion resulting from t(10;11)(p13;q21) has been reported to be associated with particularly poor survival in pediatric T-ALL cases [58].

#### 2.2.3. NOTCH1 Mutations

*NOTCH1* is a transmembrane heterodimeric receptor composed of two subunits, which is crucial for T-cell fate and differentiation. Insertion and deletion mutations are observed in more than 60% of T-ALL cases, causing constitutive activation of *NOTCH1* signaling [59]. Activated *NOTCH1* signaling leads to a massive expansion of immature T cells, consequently increasing the risk of additional leukemia lesion acquisition [60]. Moreover, constitutive activation of *NOTCH1* signaling might affect other signaling pathways including cell cycle and *NF-κB* signaling [61]. In addition, the inactivating mutations in the gene that encodes the tumor-suppressor *FBXW7* are also commonly observed in T-ALL patients, which regulates the proteasome-mediated deregulation of *NOTCH1*, resulting in the loss of *NOTCH1* protein deregulation and subsequent activation of *NOTCH1* signaling [62]. Although patients with *NOTCH1*-mutant T-ALL have favorable outcomes with standard chemotherapy, the high frequency of *NOTCH1* mutations in T-ALL has inspired significant efforts to develop new treatment protocols to improve outcomes.

#### 2.2.4. Early Thymic Precursor ALL

The early thymic precursor or early T-cell precursor (ETP) ALL occurs in 10–15% of pediatric T-ALL [63]. Recently, genetics studies have shed new light on the biology of ETP-ALLs, which are characterized by a distinct immunophenotype and a gene expression signature indicative of a very early arrest in T-cell development. Coustan-Smith et al. recognized the immunophenotypes of ETP-ALL, which is characterized by the absence of CD1a and CD8 expression, weak CD5, and expression of at least one or more of the following myeloid or stem-cell markers: CD117, CD34, human leucocyte antigen (HLA)-DR, CD13, CD33, CD11b, or CD65 [63]. This immunophenotype signature distinguishes ETP-ALL from all other T-ALL subtypes, including early T-ALL, late cortical T-ALL, and mature T-ALL (none-ETP-ALL). Coustan-Smith et al. reported that ETP-ALL is associated with high rates of chemoresistance, relapse, and dismal clinical outcomes [63]. However, Patrick et al. demonstrated that the rates of 5-year EFS and OS do not differ significantly between ETP-ALL and non-ETP-ALL in a study using a risk-adapted approach with intensified initial treatment [64]. ETP-ALL has been reported to have frequent activating mutations in RAS pathway, cytokine receptor signaling genes, IL7R pathway genes, and histone modification genes [65]. The *LMO/LYL1* and *TLX3*-mutated subgroups have a higher prevalence of ETP-ALL cases [66], and ETP-ALL patients show a similar mutational profile to that of AML patients, with hematopoietic stem-cell like gene expression profiles [67,68]. This evidence suggests that ETP-ALL might arise in very early progenitor cells with multi-lineage potential. Zhang et al. verified that ETP-ALL patients harboring mutations in the Polycomb repressor complex 2 (PRC2) core components have poor prognosis, particularly in *EZH2*-mutated cases. It is estimated that 60% of these patients relapse within 5 years [65]. Further studies and novel targeted regimens are needed to improve survival in patients with ETP-ALL based on their individual mutational profile.

#### 2.2.5. Epigenetic Abnormalities in T-ALL

Mutations in epigenetic modifiers can change the accessibility of certain parts of chroma tin to transcription factors. If this mutation occurs at the wrong stage of T cell maturation, abnormal gene expression will occur, which leads to the pathogenesis of T-ALL. The epigenetic regulators most frequently reported to be involved in T-ALL, are *PHF6*, *KDM6A*, and the member of *PRC2*, such as *EED*, *EZH2*, and *SUZ12* [69].

The recurrent mutated genes encoding chromatin modifiers and epigenetic regulators have a higher incidence among *TLX3*-positive and *TLX1*-positive cases, and particularly inactivating mutations of the gene encoding the plant homeodomain-like finger family member *PHF6* were reported to occur in approximately 16% of pediatric and 33% of adult T-ALL cases [70]. *PHF6* inactivation is often associated with genetic abnormalities of the *JAK/STAT* pathway members, such as *IL7R*, *JAK1*, *JAK3*, and *STAT5B* [71].

*KDM6A* (also termed *UTX*) is an H3K27me3 histone demethylase that functions as a tumor suppressor gene. In addition, the loss-of-function mutations of *KDM6A* occur in approximately 5–15% of T-ALLs [72,73]. The core components of the *PRC2* complex, including *EZH2*, *EED*, and *SUZ12*, are epigenetic modulators that mediates the methylation of H3K27. The reduction or abolishment of PRC2 activity results in the decreased level of H3K27 methylation. Interestingly, Roels et al. recently reported an association between *PRC2* deficiency and a hypermethylation profile [74]. Mutations or deletions of at least one of the three PRC2 members occur in 25–30% of T-ALL cases, especially in immature T-ALL. The epigenetic alteration has been reported to be associated with poor response to chemotherapy [75,76]. Some of the above-mentioned mutations are enriched in particular genomic subtypes and/or T cell developmental stages. Further studies should focus on the role of these mutations in relapse, progression and targeted therapy of T-ALL.

## 3. Genetics and Epigenetics in Relapsed ALL

Despite the cure rates exceeding 90% in pediatric ALL, it remains a pivotal cause of morbidity and mortality in children, the outcome of children with relapsed ALL is poor. Therefore, it would be extremely beneficial if more new biomarkers could be identified to predict relapse of ALL at diagnosis.

### 3.1. Genetics of Relapse

Some gene mutations have been reported to be enriched at relapse of B-ALL, such as the histone acetyltransferase gene *CREBBP*, the histone methyltransferase gene *SETD2*, and the steroid receptor gene *NR3C1* and *NR3C2* [77]. These mutations confer chemotherapy resistance and might have implications for therapeutic decisions and disease monitoring. Monitoring for the emergence of relapse-associated mutations or monitoring the dynamics of mutations clearance during induction therapy will help us to identify those patients who might benefit from early modification of therapy. Further studies will identify more relapse-associated mutations to guide therapeutic decisions [7].

### 3.2. DNA Methylation as a Biomarker to Predict Relapse of ALL

Several studies have attempted to use DNA methylation signatures to predict relapse of ALL at diagnosis. The DNA methylation patterns underlying MLL-rearranged ALL in infants have been explored, and distinct promoter CpG island methylation patterns separated different genetic subtypes. The researchers found that MLL translocations t(4;11) and t(11;19) were characterized by extensive methylation, whereas infant ALL with t(9;11) and wild-type MLL epigenetically resembled normal bone marrow. Additionally, the degree of promoter hypermethylation among infant ALL patients carrying t(4;11) or t(11;19) appeared to affect relapse-free survival and predicted a high risk of relapse [78]. Milani et al. identified 20 individual genes with DNA methylation levels that predicted relapse of ALL in 416 genes in cells from 401 children diagnosed with ALL [79]. The CpG island methylator phenotype (CIMP) is defined by extensive DNA hypermethylation of cytosines with CGIs, and CIMP status can be divided into CIMP+ or CIMP- for high or low DNA methylation levels, respectively. It has been reported that T-ALL patients with CIMP- had a significantly worse outcome compared to CIMP+ cases [80]. More importantly, CIMP classification appears to predict relapse independently of MRD, though the pattern was observed in relatively small T-ALL sample sets [79]. One common finding in most of these studies is that the children with lower methylation levels at diagnosis were more likely to relapse compared to the patients that escaped relapse [9].

### 3.3. Histone Modifications in Relapsed ALL

Combined with posttranslational modifications of histone proteins and DNA constitute the chromatin of each cell and play a pivotal role in temporal and cell-specific regulations of gene expression. Meanwhile, dynamic modification of chromatin, which results from the interaction of histone marks and DNA methylation, may contribute to the malignant transformation of normal hematopoietic precursor cells into ALL cells. However, chemical modifications of histone proteins as epigenetic marks have been less studied than DNA methylation, especially in ALL. In addition, these important and extensively described histone protein modifications include histone lysine acetylation, histone lysine methylation, and histone phosphorylation.

Histone acetylation regulated by histone lysine acetyltransferases (KATs) and histone deacetylases (HDACs) is involved in gene transcription, chromatin structure, and DNA repair, which are basic cellular phenomena in physiology and in cancers [81,82]. CREBBP is a histone acetyltransferase that can acetylate various residues in several histones, particularly in histone H3 lysine 18 (H3K18) [83]. *CREBBP* mutations and deletions were shown to be very common in relapsed cases of B-ALL (18.3% of patients). Mar et al. subsequently reported a similar frequency of *CREBBP* gene mutations in pediatric relapsed ALL cases [84]. Several studies have reported that *CREBBP* mutations are particularly prevalent in high hyperdiploid ALL [85,86]. Several HDACs have been proved to be expressed at higher levels in ALL than in normal bone marrow cells, including HDAC1, HDAC2, HDAC3, HDAC4, HDAC6, HDAC7, HDAC8, and HDAC11. Among them, expression of HDAC1, HDAC2, HDAC4, and HDAC11 is associated with unfavorable prognostic factors [87]. Sonnemann et al. demonstrated that leukemic cells from ALL cases are characterized by increased histone deacetylase activity as compared to normal bone marrow cells [88].

Methylation of various lysine residues of histone proteins is regulated by histone lysine methylases and demethylases. Several histone methyltransferases were reported to play an important role in the pathogenesis of B-ALL, especially KMT2A. KMT2A is a histone H3 lysine 4 (H3K4) methyltransferase and the methylation of H3K4 is typically associated with transcriptional activation and euchromatin [83]. *KMT2A* rearrangements are a prototypical example of leukemia driven by deregulation of the epigenetic process, which disrupt the normal function of *KMT2A* by a fusion protein partner [9]. In addition, the *KMT2A* fusion protein is regarded as a powerful cancer driven gene [33], the most common *KMT2A* rearrangement is the *KMT2A-AF4* fusion gene resulting from the translocation t(4;11)(q21;q23) in infant-ALL. Recent studies suggest that H3K79 methylation profiles are more consistently associated with *MLL1*-rearranged leukemia than H3K4 methylation profiles, and suppression of the H3K79 methyltransferase DOT1L inhibit the expression of critical *MLL1-AF4* target genes [89,90]. Other histone methyltransferases implicated in leukemogenesis of B-ALL include nuclear receptor-binding SET domain protein 2 (NSD2) [91], SET domain-containing protein 2 (SETD2), and histone lysine N-methyltransferase EZH2. NSD2 and SETD2 are both H3K36 methyltransferase, and mutations of the latter are reported in B-ALL at a relatively high frequency (12% of the entire cases). A recent study reported the frequency of *SETD2* gene mutations increased in *MLL1*- and *ETV6-RUNX1* rearranged cases, particularly increased in relapsed cases [84]. Schafer et al. found a relatively low frequency (1.3%) of mutation in *EZH2* in ALL [92], and *EZH2* gene mutations might be enriched in hypodiploid ALL [42].

Histone phosphorylation plays an important role in transcription, chromatin condensation, mitosis, apoptosis, and DNA replication [83]. Aberrant phosphorylation of several histone proteins and mutations in genes involved in histone phosphorylation are reported in multiple cancers, but there is a lack of such reports in ALL. Janus kinase (JAK) is a site of recurrent rearrangements in ALL, and JAK2 was recently reported to be able to phosphorylate histone H3 at tyrosine 41 (H3Y41), which results in dissociation of some effector proteins from chromatin [93]. Other than that, there are no studies reporting on mutations or rearrangements involved in histone phosphorylation, further studies are needed to prove the associations of histone phosphorylation markers and ALL.

## 4. Molecular Targeted Treatment of ALL

With the growing understanding of genetic alterations in ALL, approaches targeting the driving genetic mutations and/or the associated signaling pathway are emerging. Molecularly targeted therapy has been introduced in ALL treatment regimens for Ph+ B-ALL. Apart from tyrosine kinase inhibitors for Ph+ ALL, there have been no new FDA approvals for molecular targeted agents for ALL over the past decades [2]. However, there are many agents with novel molecular targets in clinical trials and at various stages of preclinical development [94].

### 4.1. Ph+ B-ALL Targeted Therapies

Ph+ B-ALL is the most successful example of how genetic alterations in B-ALL can be targeted therapeutically by small molecules. Daley et al. demonstrated that expression of the *BCR-ABL1* fusion gene was transformative and leads to the development of leukemia in mice [95]. Subsequently, Lugo et al. found that the tyrosine kinase activity of *ABL1* correlates with transformation and suggested that the inhibition of tyrosine kinase activity could be a potential therapeutic strategy [96]. After that, a screen of small molecular tyrosine kinase inhibitors identified imatinib as an ABL1 tyrosine kinase inhibitor [97]. Imatinib has been used in the treatment of CML and Ph+ ALL patients and has dramatically improved patient survival. Druker et al. showed that single-agent imatinib is curative in a high percentage of patients with CML in clinical trials. In addition, they also found that the combination of imatinib with chemotherapy in Ph+ ALL significantly improves survival [98,99]. However, some patients developed resistance to imatinib and approximately 50% of resistance results from the emergence of point mutations in the BCR-ABL1 tyrosine kinase domain which disrupts the interactions with imatinib [100]. Then second-generation ABL1 kinase inhibitors such as dasatinib and nilotinib are available, and a randomized study showed that pediatric patients who received chemotherapy with dasatinib had better EFS, overall survival (OS), and CNS disease control when compared to patients who received imatinib [35]. A third-generation inhibitor ponatinib has potent activity in both wild-type and mutant *BCR-ABL1* ALL, including *ABL1 T315I* mutation [101]. A single-center phase 2 clinical trial indicated that the combination of chemotherapy with ponatinib resulted in excellent 2-year EFS in adults with newly diagnosed Ph+ ALL [101]. However, the safety of ponatinib in combination with pediatric regimens should be evaluated in the future due to the potential adverse effects, such as thrombosis and pancreatitis.

### 4.2. Ph-like ALL Targeted Therapy

Ph-like ALL is characterized by a gene-expression profile similar to that of *BCR-ABL1*-positive ALL without *BCR-ABL1* fusion protein expressed from the t(9;22)(q34;q11.2). In addition, deletions or mutations of *IKZF1* (encoding Ikaros) are a hallmark of both Ph+ ALL and Ph-like ALL [102,103]. Transcriptome sequencing and whole-genome sequencing in fifty children with Ph-like ALL identified chromosomal rearrangements or sequence which deregulate cytokine receptor and tyrosine kinase genes [104]. Furthermore, Weston and Lengline respectively reported that patients with refractory Ph-like ALL and the *EBF1-PDGFRB* fusion have a remarkably good response to the therapy with tyrosine kinase inhibitors [105,106]. Then Roberts et al. reported that Ph-like ALL increased in frequency from 10% among children with standard-risk ALL to 27% among young adults with ALL and was associated with a poor outcome [40]. They identified several kinase-activating alterations in 91% of patients with Ph-like ALL, mutations involving *FLT3*, *IL7R*, and *SH2B3* and rearrangements involving *ABL1*, *ABL2*, *CRLF2*, *CSF1R*, *EPOR*, *JAK2*, *NTRK3*, *PDGFRB*, *PTK2B*, *TSLP*, and *TYK2* were most common. Then they demonstrated that human leukemic cells expressing *ABL1*, *ABL2, CSF1R* and *PDGFRB* fusions were sensitive to dasatinib in vitro, *EPOR* and *JAK2* rearrangements were sensitive to the JAK2 inhibitor ruxolitinib, and the *ETV6-NTRK3* fusion was sensitive to the ALK inhibitor crizotinib [40]. Subsequently, Tanasi et al. reported that patients with ABL-class kinase rearrangement show promising MRD response and outcome when exposed to tyrosine kinase inhibitor frontline or at relapse in a large cohort study [107]. Therefore, more clinical trials are needed to assess whether adding tyrosine kinase inhibitors to current therapy will improve the survival of patients with Ph-like ALL.

## 5. Epigenetic Targeted Treatment of ALL

### 5.1. KMT2A-Rearranged ALL Targeted Therapies

*KMT2A* is a DNA-binding histone methyltransferase to epigenetically regulate gene expression in a multiprotein complex. The oncogenic *KMT2A* fusion protein loses the histone methyltransferase domain and is fused to a large number of partner proteins, leading to abnormal function. *KMT2A* rearrangement ALL is increasingly recognized to be driven by aberrant epigenetic programs. The novel therapeutic strategies targeting *KMT2A* rearrangement ALL are emerging.

The H3K79 methyltransferase DOT1L is a required component of the aberrant epigenetic state and *KMT2A*-rearranged leukemogenesis. Daigle et al. demonstrated that highly selective small molecule inhibitors of DOT1L had promising activity in preclinical models of *KMT2A*-rearranged leukemia [88]. However, the clinical activity of the first DOT1L inhibitor studied, pinometostat, was limited when used as monotherapy in relapsed children and adults with *KMT2A*-rearranged leukemia in a Phase 1 study [108].

*BRD4* (bromodomain containing 4) binds acetylated histones and facilitates transcription downstream of *MYC* and other validated oncogenes. Zuber et al. identified that BRD4 is required for the maintenance of leukemia in an MLL-AF9 murine model through a non-biased RNA interference screen of 243 chromatin modifying genes [109]. Subsequently, Dawson et al. demonstrated that selective small inhibitors of *BRD4* downregulated *KMT2A*-rearranged and *MYC* target genes and proved antileukemic activity by inducing apoptosis and differentiation in vitro and in vivo [110].

Another characteristic of *KMT2A*-rearranged ALL is the epigenetic silencing of another set of tumor-suppressor genes through hypermethylation of the promoter region CpG island [111]. Moreover, Stumpel et al. found that increasing degrees of promoter hypermethylation correlated with inferior survival [77]. Several studies have demonstrated that demethylating agents such as azacytidine, decitabine, and zebularine preferentially reverse aberrant DNA methylation and effectively induce apoptosis in *KMT2A*-rearranged ALL cells [77,112,113].

In addition to the above targeted therapies, studies about some other agents are also ongoing. Seyfried et al. verified that venetoclax can inhibit the anti-apoptotic regulator *BCL-2* and deregulated cell death pathways contribute to treatment failure in ALL [111]. Preclinical studies have identified the activities of venetoclax against high-risk leukemias such as *KMT2A*-rearranged ALL, ETP-ALL, and hypodiploid ALL [114,115]. Proteasome inhibitor, bortezomib, and mTOR inhibitors have also been proved to produce efficacy in children with relapsed ALL [116,117].

### 5.2. Targeting Histone Modifications in ALL

Epigenetic alterations are relatively susceptible to small molecule agents compared to genetic mutations. Histone deacetylase inhibitors (HDACi) are a class of drugs that can alter the epigenetic state of ALL cells. Several preclinical and clinical studies have examined HDACi as potential therapeutic agents in ALL. Currently, the HDACi vorinostat has been approved for the treatment of cutaneous T-cell lymphoma [118].

HDACi have been shown to induce cell cycle arrest, terminal differentiation, and/or apoptosis in vitro and animal models of ALL. Several preclinical studies have shown promising results in hematological malignancies, especially in ALL (Table 2). Several clinical trials are ongoing to assess HDACi as potential therapies for ALL. However, HDACi are less effective and more toxic in vivo than they appeared to be in vitro. Burke et al. reported that the toxicity of the combination of decitabine and vorinostat was not acceptable in children with relapsed/refractory B-ALL but did demonstrate potent pharmacodynamic modulation of biological pathways associated with anti-leukemic effects [119]. We need more studies to find safe and effective regimens in clinical applications of ALL.

The combination of epigenetic targeted therapy with CD19-targeted immunotherapy may be another strategy for reducing the intensity of myelosuppressive chemotherapy required to induce a clinical response in children with ALL. Further studies are needed to determine whether epigenetic modification therapies can be successfully combined with multi-agent chemotherapy and other therapies to treat children with multiple relapsed leukemia.

### 5.3. Other Potential Epigenetic Targeted Therapy

DNA methylation is another major epigenetic modification, and DNA methyltransferases inhibitors have been applied in other hematologic malignancies, such as AML and MDS [120]. Azacitidine and decitabine are nucleoside analogous, which are incorporated into DNA leading to depletion of DNMTs, hypomethylation of DNA, and induction of DNA damages [121]. Both DNA methyltransferases inhibitors have been approved for the treatment of MDS as well as AML [122]. Sun et al. proved that azacitidine followed by intensive chemotherapy could be applied to treat children with relapsed or refractory AML [123]. However, they found that neither of the patients with ALL responded with azacitidine [124]. Lu et al. suggested that decitabine enhances chemosensitivity in both cell lines and patient-derived samples of ETP-ALL [122]. Further studies are required to enhance our understanding of whether azacitidine or decitabine can be the candidate drugs in ALL.

Compared to AML, the current therapeutic approaches for ALL do not include regimens targeting epigenetic modifications, which might result in the relapse in some pediatric cases, and combine epigenetic therapies with conventional chemotherapy and immunotherapy might improve the safety and efficacy for ALL cases with specific genetic and epigenetic characteristics.

## 6. Conclusions and Future Perspectives

An increasing number of studies have proved that epigenetic abnormalities in pediatric ALL play an important role in the development of ALL, disease progression, and relapse. Combined with the heterogeneity of the cytogenetic subtypes of ALL, the characteristic patterns of DNA methylation and other epigenetic features make almost every patient unique. Major advances in the genetic and epigenetic profiles of ALL improve the risk stratification of patients, and epigenetic abnormalities, especially DNA methylations, are excellent biomarkers for ALL for stratifying patients and predicting relapse.

The studies described above emphasize the importance of epigenetic control in leukemogenesis, and the increasing genetic and epigenetic studies in the understanding of ALL pathogenesis is providing more opportunities for drug development. Many potential targets have been identified in ALL, including oncogene fusion proteins, cell surface antigens, kinases, epigenetic regulators. In addition, the great success of tyrosine kinase inhibitors for Ph+ ALL implicate the enormous potential of developing genetic and epigenetic targeted approaches.

Further studies should pay more attention to the full spectrum of genetic and epigenetic alterations in ALL through large-scale sequencing, then we could know more about the detailed mechanisms of disease pathogenesis, so as to uncover new potential targets. Meanwhile, more preclinical and clinical studies of these targeted therapies will provide more data to guide treatment strategies in order to achieve maximal clinical benefit with minimal toxicity. Nowadays, immune-based therapeutic agents (including monoclonal antibodies, chimeric antigen receptor-T cells, and bispecific T cell-engaging antibodies) have been proven to be an exciting and promising area. The genomic background could influence response to immunotherapy through different combinations of mutations. Further studies are warranted to identify the potential relationship between co-occurring mutations and response to immunotherapy. Some studies have demonstrated that epigenetic therapies, such as HDACi and DNMTi, are promising therapeutic agents for reversing tumor immune resistance and sensitizing tumors to immune therapy in a wide variety of solid tumors [125]. We believe that harnessing the potential of epigenetic therapies to reverse tumor immune resistance and sensitize to curative immune therapy may also be a safe and effective approach in ALL in the future.

## Figures and Tables

**Table 1:** Genetic alterations and potential targeted therapy in pediatric B- and T-acute lymphoblastic leukemia.

Classification	Frequency	Prognosis	Potential Therapeutic Implications
**B-cell acute lymphoblastic leukemia**			
High hyperdiploidy (HeH)	~25% of pediatric ALL	Excellent prognosis	Reduction of intensity
Hypodiploidy	~1–2% of ALL	Inferior survival	BCL2 inhibitors
t(12;21)(p13;q22) encoding *ETV6-RUNX1*	~25% of standard risk pediatric B-ALL	Excellent prognosis	Reduction of intensity
*ETV6-RUNX1*-like		Favorable prognosis	Reduction of intensity
*KMT2A* (*MLL*) rearranged	~75% of infants with B-ALL	Dismal survival	DOT1L inhibitors, menin inhibitors, proteasome inhibitors, HDAC inhibitors, BCL2 inhibitors
t(9;22)(q34;q11.2) encoding *BCR-ABL1*	3–5% of pediatric B-ALL	Historically poor prognosis, improved with tyrosine kinase inhibitors	ABL1 inhibitors, FAK inhibitors, rexinoids, BCL2 inhibitors
t(1;19)(q23;p13.3) encoding *TCF3-PBX1*	4% of ALL	Favorable prognosis	
iAMP21	~2% of pediatric B-ALL, older children	High-risk therapy for good outcomes	Intensification of therapy
Ph-like	12–15% of pediatric B-ALL	Poor survival	ABL1 inhibitors, JAK inhibitors, PI3K inhibitors, BCL2 inhibitors
*DUX4* rearranged	7% of childhood B-ALL	Favorable prognosis	Reduction of intensity
*MEF2D* rearranged	3–6% of childhood B-ALL	Poor survival	HDAC inhibitors
*ZN384* rearranged	3% of childhood B-ALL	Intermediate prognosis	FLT3 inhibitors
*NUTM1* rearranged	1–2% of pediatric B-ALL	Excellent prognosis	HDAC inhibitors, bromodomain inhibitors
**T-cell acute lymphoblastic leukemia**			
*NOTCH1* mutation	>50% of childhood T-ALL	Favorable outcomes	Standard chemotherapy
*TAL1* deregulation	30% of childhood T-ALL	Enrichment of mutations in PI3K signaling pathway	PI3K inhibitors, nelarabine, BCL2 inhibitors
*TLX3* deregulation	19% of childhood T-ALL	Poor prognosis	Nelarabine, BCL2 inhibitors
*HOXA* deregulation	5% of childhood T-ALL	Frequent mutations in JAK-STAT pathway, *KMT2A* rearrangements	JAK inhibitors, nelarabine, BCL2 inhibitors
*TLX1* deregulation	8% of T-ALL	Favorable prognosis	Nelarabine, BCL2 inhibitors
*LMO2/LYL1* deregulation	13% of childhood T-ALL	Poor prognosis	JAK inhibitors, nelarabine, BCL2 inhibitors
*NUP214-ABL1* with 9q34 amplification	~5–10% of childhood T-ALL	Neutral prognosis	ABL1 inhibitors, nelarabine, BCL2 inhibitors
*NKX2-1* deregulation	8% of T-ALL	Frequent co-operating mutation in ribosomal genes	Nelarabine, BCL2 inhibitors
Early T-cell precursor ALL	10–15% of T-ALL	Poor prognosis	JAK inhibitors, BCL2 inhibitors

**Table 2:** Clinical trials targeting or potentially targeting histone modifications in ALL.

NCT Number	Phase	Epigenetic Therapy	Target	ALL Conditions	Start Year	Status
NCT00053963	I	FR901228	Histone deacetylases	Refractory (0–21 years)	2002	Completed
NCT00217412	I	Vorinostat	Histone deacetylases	Relapsed or refractory (1–21 years)	2005	Completed
NCT00882206	II	Vorinostat	Histone deacetylases	Relapsed or refractory (2–60 years)	2009	Completed
NCT01251965	I/II	Ruxolitinib	JAK1/JAK2 kinases	Relapsed or refractory (14 years, or older)	2010	Completed
NCT01321346	I	Panobinostat	Histone deacetylases	Refractory (8–21 years)	2011	Completed
NCT02141828	I	EPZ-5676	H3K79 methyltransferases	Relapsed or refractory (0–18 years) MLL-rearranged	2014	Completed
NCT02419755	II	Vorinostat	Histone deacetylases	Relapsed or refractory (0–21 years) MLL-rearranged	2015	Completed
NCT02420717	II	Ruxolitinib	JAK1/JAK2 kinases	Ph-like (10 years or older)	2015	Completed
NCT02723994	II	Ruxolitinib	JAK1/JAK2 kinases	CRLF2-rearrange and/or JAK pathway-mutant (1–21 years)	2016	Recruiting
